# In Silico Design of a Peptide Receptor for Dopamine Recognition

**DOI:** 10.3390/molecules25235509

**Published:** 2020-11-25

**Authors:** Luna Rodriguez-Salazar, James Guevara-Pulido, Andrés Cifuentes

**Affiliations:** 1 Bioingeniería, Universidad El Bosque, Bogotá 110121, Colombia; lrodriguezsal@unbosque.edu.co; 2 Química Farmacéutica, Facultad de Ciencias, Universidad El Bosque, Bogotá 110121, Colombia; accifuentesl@correo.udistrital.edu.co

**Keywords:** in silico, dopamine, molecular docking, molecular dynamics, bioreceptor

## Abstract

Dopamine (DA) is an important neurotransmitter with a fundamental role in regulatory functions related to the central, peripheral, renal, and hormonal nervous systems. Dopaminergic neurotransmission dysfunctions are commonly associated with several diseases; thus, in situ quantification of DA is a major challenge. To achieve this goal, enzyme-based biosensors have been employed for substrate recognition in the past. However, due to their sensitivity to changes in temperature and pH levels, new peptide bioreceptors have been developed. Therefore, in this study, four bioreceptors were designed in silico to exhibit a higher affinity for DA than the DA transporters (DATs). The design was based on the hot spots of the active sites of crystallized enzyme structures that are physiologically related to DA. The affinities between the chosen targets and designed bioreceptors were calculated using AutoDock Vina. Additionally, the binding free energy, ∆G, of the dopamine-4xp1 complex was calculated by molecular dynamics (MD). This value presented a direct relationship with the E_refine value obtained from molecular docking based on the ∆G functions obtained from MOE of the promising bioreceptors. The control variables in the design were amino acids, bond type, steric volume, stereochemistry, affinity, and interaction distances. As part of the results, three out of the four bioreceptor candidates presented promising values in terms of DA affinity and distance.

## 1. Introduction

Dopamine (DA) is a catecholamine that can act as a hormone or neurotransmitter [1] due to its functions in the central nervous system and peripheral system as well as in other tissues, such as the gastrointestinal and renal tissues [2]; it also functions as a sodium regulator [3]. Furthermore, its importance in motor control, learning, motivation, memory, and in the brain’s reward system has also been described [4]. Because DA plays a critical role in the regulation of vital functions, dopaminergic neurotransmission dysfunctions are commonly associated with multiple disorders [5], such as schizophrenia, Parkinson’s disease [6] and Alzheimer’s disease [7].

The importance of DA as a neurotransmitter extends beyond its relationship with diseases since it is also used as a diagnostic tool [8]; for example, catecholamines detected in urine samples, are tumor markers for neuroblastomas and pheochromocytomas.

Hence, this evidences the need for reliable bioanalytical methods for the extraction, separation, and quantification of these diagnostic markers [1]. Nevertheless, it must also be considered that catecholamines are unstable, prone to spontaneous oxidation, can easily decompose at high pH levels [9], and are also found at low concentrations in biological samples needing rigorous pretreatment steps for an interference-free quantification [3]. Therefore, DA quantification methods are being researched, and their mechanisms of action and ligands of interaction with nervous system receptors are also being assessed using in silico models [10]. This study is conducted because DA is considered as one of the most important neurotransmitters in mammals [8].

There are currently new in vitro techniques for the quantification of DA; however, the standard method used is high-performance liquid chromatography with electrochemical detection (ECD) [11]. Considering the above-mentioned difficulties, these techniques do not yield reproducible results in real time or in situ analysis. Therefore, biosensors have emerged as a tool capable of potentially offering a real-time highly selective and sensitive detection of the substrate [12].

The DA biosensors proposed to date use the tyrosinase enzyme as the bioreceptor (EC 1.14.18.1) [13]. However, enzymatic bioreceptors exhibit their own disadvantages, such as low stability against pH and temperature variations, and critical operating conditions, which significantly hinders their usage in real-time analysis [14]. Consequently, new unconventional bioreceptors have been designed for the recognition of substrates, representing a fundamental step in the development of new diagnostic tools [15]. For example, peptides are emerging as an alternative for molecular recognition, as they are considered versatile molecules because they can be synthesized with a wide variety of structural modifications [16,17]. In silico models of peptide bioreceptors [18] and peptide-based biosensors have already been designed for substrate recognition [19].

Therefore, our in silico study focuses on designing bioreceptors capable of recognizing a particular substrate [18], in this case DA, as a previous step to synthesizing the most promising bioreceptors obtained. This way, the in vitro design and testing process can be more efficient and faster due to the implementation of bioinformatics tools, such as molecular modeling, to assess and improve the bioreceptor–ligand interaction compared to that of the protein–ligand complex interaction [20] between DA and the DA transporter (DAT) during dopaminergic neurotransmission [21].

## 2. Results

The results of this study are presented below in four sections. The first section presents the targets chosen due to their association with DA and the interaction energy resulting from each target–DA complex. The second section presents the control variables of the design of the bioreceptors and its affinity with DA after each structural modification. The third section includes the description of the parameters that allowed three bioreceptor candidates to be selected for future synthesis as well as the molecular interaction distances between DA and each bioreceptor. Finally, the fourth section analyzes the molecular dynamics of each dopamine–bioreceptor complex

### 2.1. Selection and Analysis of Targets and Their Interaction with DA

#### 2.1.1. Target Selection

According to the methodology proposed and followed, enzymes belonging to the enzyme class 1.14.18 were retrieved from the Protein Data Bank (PDB). However, the results only yielded the following three crystallized enzymes: tyrosinase (1.14.18.1) as 5M8L, methane monooxygenase (particulate) (1.14.18.9) as 1YEW, and methylsterol monooxygenase (1.14.18.9) as 4IIT.

Since only three crystallized enzymes were found out of the nine enzymes proposed, the authors decided to seek other targets that interact with DA or its analogs in physiological processes as well as those crystallized using DA as the ligand. Accordingly, the following 15 targets were found (Table 1).

#### 2.1.2. Analysis of Target–DA Interactions

Table 2 presents the docking results obtained for each target, calculated as the average of the three docking runs. Based on these results, the four enzymes selected with the highest affinity for DA were 5M8L, 2A3R, 4IIT, and 2E82 which are in bold text in Table 2. These enzymes will be used as a starting point for the bioreceptor design.

The sequences of each target were analyzed in the PDB. Such analysis was based on determining the nature of each enzyme’s active site, such as its dimensions, and identifying the hotspots of the active site that interact with LDP (L-Dopamine). Afterward, in AutoDock Tools, those amino acids were selected, and the dimensions and coordinates of the grid box were adjusted to ensure that every amino acid was inside the grid. With these dimensions and coordinates, docking in AutoDock Vina was performed to calculate their interaction energy with DA.

### 2.2. Design and Analysis of Bioreceptors that Interact with DA

The bioreceptors were designed to be miniature versions of DAT, the protein responsible for DA reuptake into the presynaptic neurons [22], but with better affinity and molecular distances. Considering that this protein is activated or deactivated according to both the short- and long-term physiological demands of the neurons [22], the bioreceptors must mimic its ability to recognize the DA present at the synaptic cleft when coupled to the biosensor system. 

It is important to mention that miniaturization is a concept that has been defined as “the process of doing something on a very small scale using modern technology” [23] or as “a version of something on a small scale or small size” [24]. In addition, this miniaturization trend was first applied to electronic devices in the 1960s and later evolved to biological molecules [25] and drug release mechanisms [26].

It is important to keep in mind that human DAT (hDAT) has not been crystallized yet; therefore, there is no information about its crystal structure determined through experimental methods. However, the structure of the DATs of other species, such as *Drosophila melanogaster*, has already been determined [27] (the PDB code for this protein is 4XP1), and it has been described that the 4XP1 protein is homologous to hDAT. However, there are computational models that have simulated the structure and interactions of hDAT [28], and this information will also be considered in the design of proposed bioreceptors.

Furthermore, the initial bioreceptors were designed based on the amino acids of the active site of the 5M8L, 4IIT, 2A3R, and 2E82 enzymes that exhibit a direct interaction with the ligand, also known as the active site’s hotspots. Subsequently, structural changes were sequentially made to these initial bioreceptors as per the provisions included in the methodology.

The first group of bioreceptors was made out of peptides built based on the amino acids from the hot spots of 5M8L(1.0), 4IIT (2.0), 2A3R (3.0), and 2E82(4.0). Table 3 denotes their identification code, their amino acid sequence, their average interaction affinity after performing this calculation thrice, and the interaction affinity’s standard deviation.

As expected, the results indicated that the affinity of the three bioreceptors for DA was lower than the affinity of DAT for DA, which is −5.2 kcal/mol. As previously reported, these results were expected because the steric hindrance exhibited by a peptide within this design length range is not comparable with that of a target [18]. 

Thus, two options were considered. The first was to consider the number of residues between the selected amino acids from each hot spot, and the second was to increase the steric volume of the bioreceptors in order to increase their affinity [18].

Accordingly, Table 4 shows a new group of bioreceptors where methylene bridges were added to the previous amino acid sequences from Table 3 to preserve the distances presented within the target’s active site. This table also presents interaction affinity averages calculated as previously described with their respective standard deviation. 

In this case, if the interaction energies of the bioreceptors composed solely of peptides are compared against the interaction energies from the bioreceptors to which the methylene bridges were added, no conclusive pattern may be observed. In some cases, the energy increased (bioreceptors 1.1 and 3.1, Table 3, second and fourth row), whereas in others, it decreased (bioreceptor 2.1). However, it was considered that because DA is a small molecule and does not have many atoms that may allow it to interact with other molecules, the amino acids are distant. Therefore, for the bioreceptor 2.1, the possible interactions that existed before adding the methylene bridges were lost. This result contradicts that of a previous study [18], but if it is considered that the substrate size in the study was much larger compared to DA, it could be deduced that DA can only generate a few interactions.

**Figure 1:**
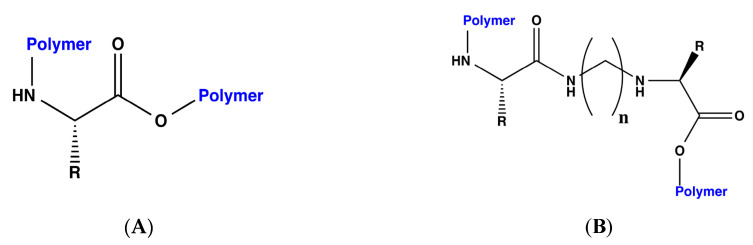
General scheme for the polymerization of the bioreceptors. (**A**) For those bioreceptors composed solely of peptides. (**B**) For those bioreceptors with methylene bridges within the amino acid sequence.

Considering that in previous studies, steric volume increased interaction energies [18], each of the designed bioreceptors, those with and without the methylene bridges, were again modified by polymerizing the C and N terminals of each peptide. The general scheme for the polymerization is shown in Figure 1. 

The following two different polymers were used for this purpose: polyethylene and polystyrene. Once designed, the interaction energy for DA was calculated for each of the bioreceptors. The results of these dockings with polyethylene and polystyrene are presented in Table 5, with their corresponding code and sequence. 

According to the results presented in Table 5 compared to those in Table 3 and Table 4, it is evident that affinity increases in all cases when the steric volume increases by means of polymerization. In this case particularly, there is a recognizable pattern. In addition, polymerization with polystyrene increases the affinity for DA in contrast to that of the bioreceptors polymerized with polyethylene. On the other hand, these results follow the pattern set out previously [18], in which it has been stated that recognition interaction improves when polymerization is performed with polystyrene.

Continuing with the design of the bioreceptors and based on the above results, we decided to study which of the variables of the amino acids could influence affinity for DA. Thus, the first criterion was stereochemistry. Except for glycine, all other amino acids have a chiral carbon, which exhibits bonds with four different atoms or groups of atoms [29], thereby generating an enantiomer pair of spatial isomers defined as non-superimposable mirror images of each other [30]. It is important to keep in mind that the amino acids’ natural stereochemistry in the human body is the L configuration and not the D configuration [29]. However, this section uses the *R* and *S* nomenclature, which applies to natural compounds and determines the stereochemistry based on the importance defined by the atomic number of the chiral carbon substituent. Using this information, amino acid stereochemistry was selected as the first variable to be analyzed to identify its influence on their interaction with DA.

To address the first variation, the NWR bioreceptor polymerized with polystyrene (Table 5; code: 1.0.2) was selected because this was the polymerization that provided the best results and because its interaction with DA was restricted to only those three amino acids. In contrast, it cannot be accurately identified which of the amino acids from the larger peptides are responsible for their interaction with DA. Similarly, from the interactions between the computational model of hDAT and DA, it has been determined that such interaction occurs only due to two or four amino acids [28]. 

**Figure 2:**
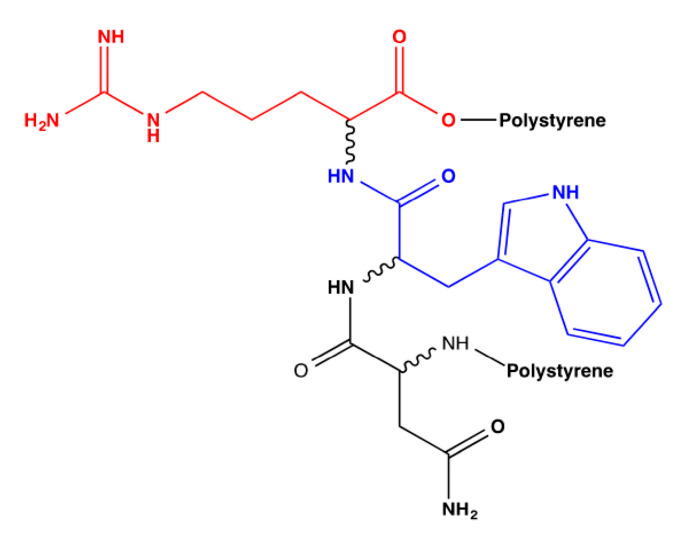
Polystyrene–NWR–polystyrene bioreceptor showing each of the amino acids that will be stereochemically modified.

Therefore, all possible combinations of the *R* and *S* amino acids of the NWR tripeptide polymerized with polystyrene were built. Figure 2 presents the flat structure of this bioreceptor, where each amino acid whose stereochemistry will be modified is identified with a different color, and Table 6 denotes the codes for the derived bioreceptors with the stereochemistry of each amino acid in its respective order and their average interaction energy in kcal/mol.

As seen in Table 6, there are variations in the affinity results for each bioreceptor according to modifications in the stereochemistry of the amino acids that compose them. Hence, it was determined that the bioreceptor that best interacts with DA is the one with SSS stereochemistry (code 1.0.2), as shown in Table 6. Although the other results were not considerably distant, this result was obtained because this is the natural stereochemistry of amino acids, and therefore, the other results exhibited decreased affinity. It is worth mentioning that the bioreceptor with SSS stereochemistry is the same one that was designed by polymerization with polystyrene, which is why the code did not change. The standard deviation of the data is generally reduced to one decimal or even becomes null in some cases, which means that the data dispersion is not very variable.

However, once we defined that we wanted to maintain the natural amino acid stereochemistry in the bioreceptor design, we proceeded to determine how many amino acids each bioreceptor should have.

**Figure 3:**
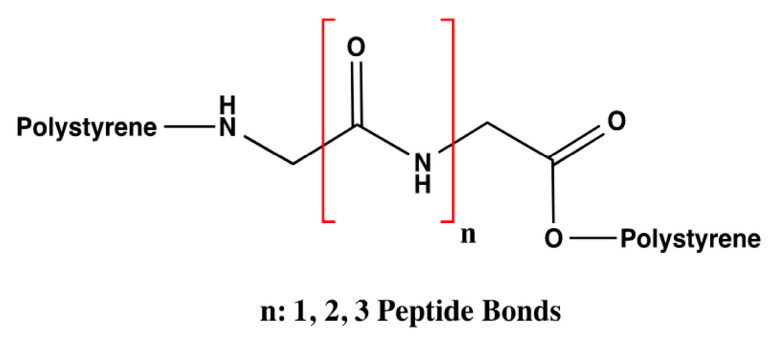
General structure of the bioreceptors with variations in the amount of glycine.

Based on the hDAT model described IN [28] and the analysis of the number of interactions that DA can form, the influence of the number of amino acids was evaluated only with three styrene-polymerized bioreceptors according to the previous results. The design of these bioreceptors was carried out only with glycine so that only the interaction of DA with the number of peptide bonds could be assessed with no influence from the functional groups that compose the substituents of the other amino acids. The number of amino acids varied from two to four glycine molecules, as shown in Figure 3.

Considering this, Table 7 indicates the affinity results for the three glycine bioreceptors, specifying the amount of glycine they contain and their average interaction energy in kcal/mol. 

As shown in Table 7, the best results were obtained with the bioreceptor composed of three glycine molecules (4.1, Table 7) with an affinity value of −5.1 kcal/mol. This value was taken as the parameter used to build the following bioreceptors in order to study the relationship of their affinity for DA according to the amino acids that compose them.

**Figure 4:**
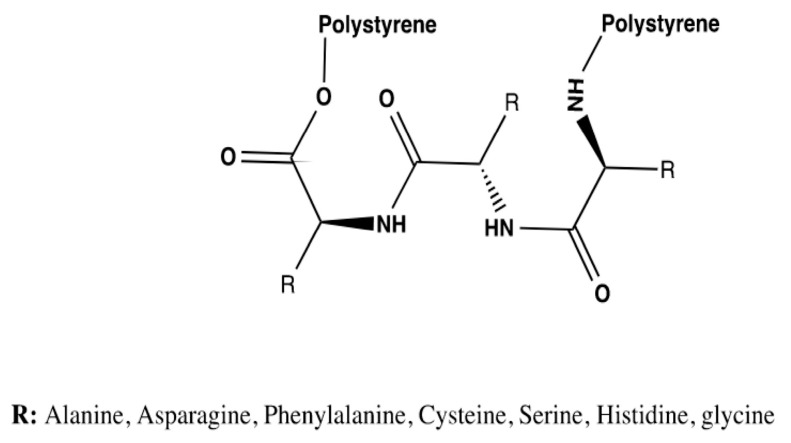
The general structure of the receptors wherein the nature of amino acids is studied.

Thus, tripeptide bioreceptors were designed to study the influence of the amino acid’s nature on the interaction energies exhibited. A total of seven tripeptide bioreceptors made out of glycine, phenylalanine, alanine, asparagine, serine, cysteine, and histidine were modeled. The general structure for this group of bioreceptors is shown in Figure 4.

Regarding the previous results, and as mentioned before, glycine was used to determine if the substituents of the amino acids that comprise the bioreceptors were essential or if the peptide bonds alone could generate enough affinity for DA. This glycine tripeptide’s affinity result is presented in the first row of Table 8 and is the same one presented in Table 7 under code 4.1.

Continuing with the variable evaluation and seeking to understand the influence of the amino acids’ nature on the non-covalent interactions of the bioreceptor, phenylalanine and alanine were initially selected as standards for the group of non-polar amino acids to simultaneously compare the influence of the amino acids with an aromatic substituent, which made it possible to analyze the π-π interaction that can occur between the amino acids themselves or with DA, as shown in Table 8. We identified that this interaction can occur with phenylalanine [31,32]. Therefore, the bioreceptor results with phenylalanine can be compared with those that are formed only by alanine, which is also non-polar, but with an aliphatic and single-carbon substituent. This allows the result to be related to the type of interaction that can be formed and to the steric volume of the amino acid.

The result of the average affinity of the bioreceptor composed of a phenylalanine tripeptide is shown in Table 8 line 2 under code 5. The alanine bioreceptor corresponds to code 6 and is presented in the third row of the same Table.

The group of polar amino acids was addressed by the bioreceptors designed with asparagine (code 7), serine (code 8), and cysteine (code 9). However, there are differences in the substituents of these three amino acids, which were considered during their selection.

Asparagine is an amino acid that, in addition to being polar, has the amide functional group (RCONH_2_) in its substituent and has the capacity to accept three and donate two hydrogen bonds [33]. On the other hand, serine has a hydroxyl group in its substituent and can donate three and accept four hydrogen bonds [34]. Cysteine is a thiol [35]. The results of these three bioreceptors are reported in rows four, five, and six, respectively, of Table 8.

Histidine is a basic amino acid because of the chain in its substituent. It was selected because of its high reactivity and because it is an amino acid that plays an important role in the catalytic activity of proteins [36]. The histidine bioreceptor was assigned code 10, and the result of its interaction energy with DA is reported in row seven of Table 8.

As denoted in Table 8, the results for this series of amino acids range from −4.3 to −5.1 kcal/mol, wherein only one of the bioreceptors has a standard deviation other than zero, which means that there was no variability between them and that in the case of bioreceptor 10, data dispersion decreased. Bioreceptor 6, composed of an alanine tripeptide, exhibited a more distant result than the others, as shown in row four of Table 8. This may mean that the alanine substituent (CH_3_) did not generate a significant affinity with DA, and this result is comparable with that of phenylalanine (row 3 of Table 8), which is also non-polar and provides better results. Therefore, these π–π interactions are stronger than those formed by alanine, as mentioned in the amino acid selection criteria.

The next lowest result found was −4.5 kcal/mol, corresponding to bioreceptor 7, presented in row five of Table 8. Here, the substituent was an amide which did not exhibit any worthwhile results despite being able to donate two and receive three hydrogen bonds. This may be because these protons possess a very weak acidic character; therefore, hydrogen bond interactions will probably be unlikely with, for example, the protons of aspartic acid [37]. These results are comparable with those of the serine and cysteine bioreceptors which are also weak despite having acidic protons.

Based on the structural modifications made and that assessing the three variables mentioned above failed to achieve a bioreceptor with better affinity results, it was considered that mixtures between the different amino acids will potentiate the results, especially since it was observed that the expected results of the glycine bioreceptor were not attained by the other bioreceptors composed only of one amino acid. Then, to combine these amino acids, we decided to use the groups of amino acids that exhibited interaction in the models of hDAT and of DA receptors, which were taken from previous studies [10,28].

Before analyzing the results obtained for this group of bioreceptors, it was necessary to highlight that it had already been determined that the number of amino acids should not exceed four to ensure that interactions with DA were specifically occurring with the amino acids of interest. The existence of aromatic amino acids showed an increase in the interaction, and to add steric volume, the polymerization had to be performed with polystyrene.

Consequently, after testing the variables above, 13 additional bioreceptors were designed which correspond to the amino acid groups identified in the computational models both of the hDAT [28] and the interaction mechanism of DA receptors [10].

Overall, it has been reported that both intra and extracellular DA interactions with DATs and DA receptors involve groups of amino acids, with the number ranging from two to four. In fact, a study argues that aspartic acid is a very important amino acid and essential for DA reuptake. In addition, several aromatic interactions were also identified as playing a prominent role in the activity of DAT with DA [28].

In total, 13 combinations of amino acids were designed. They were polymerized at the C and N terminals with polystyrene to give them steric volume, a characteristic that had already been proven to increase bioreceptor interaction. Table 9 displays the code assigned to each bioreceptor, the peptide from which it was composed, and each of their average affinity results.

When observing the results obtained for the 13 bioreceptors presented in Table 9, the affinity range obtained was determined to be ranging from −4.5 to −5.4 kcal/mol, with null or 0.1 standard deviations, indicating that data dispersion was insignificant. We were able to obtain bioreceptors with energies exceeded the interaction energy of the DAT previously stated as −5.2 kcal/mol. The two bioreceptors that showed an improved interaction with DA were 15 and 19, which are in rows five and ten in Table 9.

First of all, bioreceptor 15, denoted in row five of the previous table, will be discussed. This bioreceptor is composed of serine, aspartic acid, and tryptophan. As per the above-mentioned findings, serine is a polar amino acid because of its hydroxyl group, which has been described as playing a prominent role in the catalytic activity of enzymes [34]. Conversely, aspartic acid was not addressed or considered in the tests; therefore, it is essential to emphasize its characteristics. This amino acid is acidic and can donate three and accept five hydrogen bonds; therefore, it could be said that it is the amino acid with the highest number of interactions to date [31,38]. The final one is tryptophan, which is part of the group of non-polar and aromatic amino acids. Its substituent has an indole functional group and can donate and accept three hydrogen bonds [39].

This bioreceptor exhibited an interaction energy of –5.3 kcal/mol. Its corresponding image shows how the amino acids were exposed, and the polymer provided steric volume creating a free pocket for its interaction with DA.

Bioreceptor 19 from Table 9, composed of tryptophan, phenylalanine, and tyrosine obtained an affinity of –5.4 kcal/mol. Out of these three amino acids, tyrosine, an aromatic and polar amino acid capable of donating three and accepting four hydrogen bonds, was the only one not analyzed [40]. It is among the amino acids found at the highest percentage of protein composition and has a phenol functional group in its substituent [41].

As previously described, this bioreceptor contains a tripeptide of aromatic amino acids, which reaffirms the idea that the π–π interaction is essential for DA recognition. However, it is evident that the aromatic group is not strong enough to interact with DA alone but, when supplemented by the hydroxyl group in the tyrosine ring and the benzo-fused substituent of tryptophan, they interact together to release more energy. The image depicting this bioreceptor also shows that the peptide bonds form a curve that exposes the amino acid substituents so that they can interact with DA.

### 2.3. ∆G Free Binding Energy Calculated with Molecular Dynamics (MD)

Based on the results, the study continued with two candidates that exceeded the affinity parameters and an additional one with promising characteristics owing to the differences in the upper and lower root-mean-square deviation (RMSD) values. Next, we analyzed the ∆G binding free energy calculated for the dopamine-4xp1 complex with the MD algorithm as shown in Table 10 where we obtained a ∆G binding energy of −26.760 ± 0.8 kcal/mol. This negative value represents a high ligand–receptor affinity, as might be expected, with the *Drosophila melanogaster* protein. 

**Figure 5:**
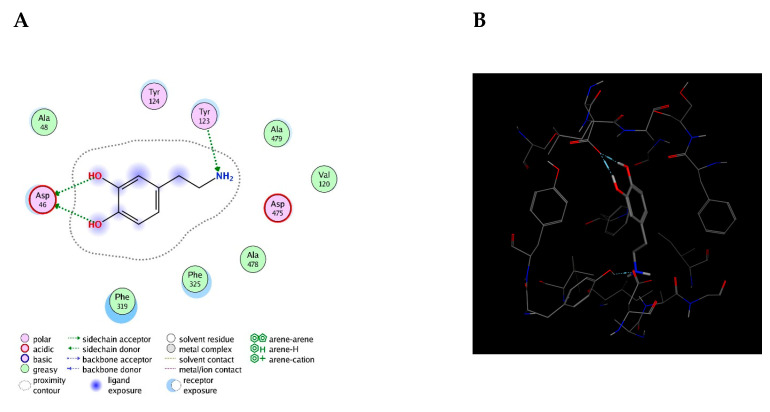
Representative ligand–complex interactions for the best score of the dopamine-4xp1 complex. (**A**) Analysis of 2D interactions. (**B**) Best score position in binding-pocket.

This value presented a direct relationship with the E_refine value obtained from molecular docking based on ∆G functions carried out with MOE as seen in Table 11, where the best s-score pose docked molecule Figure 5 obtained an E_refine of −21.43 kcal/mol. Representative non-covalent interactions are shown in Figure 5 where two important interactions stand out: 1. acid and sidechain donor interactions between residue ASP 46 and dopamine’s hydroxyl groups; 2. polar and side-chain acceptor interactions between residue Tyr 123 and dopamine’s amino group. Thus, we determined the dock pose with the best non-covalent interactions from the other dock poses obtained. Therefore, it is possible to establish that the E_refine parameter is proportionally related to the non-covalent receptor–ligand interactions.

**Figure 6:**
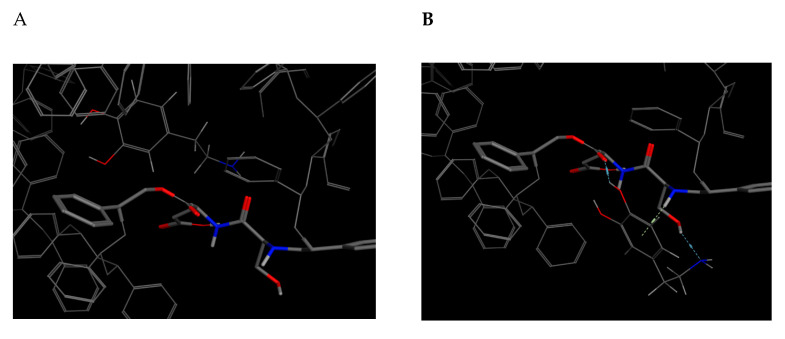
Docking score results for the dopamine–SD complex (docked with MOE). (**A**) Best score pose. (**B**) Best E_refine (molecule 2) with the best dopamine–bioreceptor non-covalent interactions.

As for the SD receptor (Table 9, code 14), of the five obtained dock poses, those with the best s-score do not present the best non-covalent interactions, as observed in Figure 6. The dock pose 2 has the best E_refine at −17.7353 kcal/mol Table 12 and the best non-covalent interactions with the SD amino acids, although it does not represent the best s-score.

**Figure 7:**
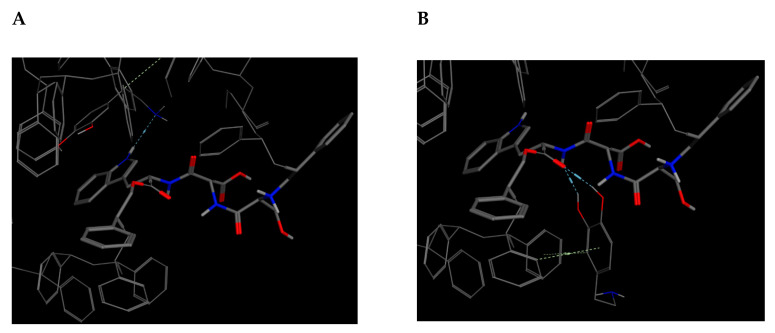
Docking score results for the dopamine–SDW complex (docked with MOE). (**A**) Best score pose. (**B**) Best E_refine (molecule 3) with the best dopamine–bioreceptor non-covalent interactions.

Similarly, to the SD bioreceptor, the SDW bioreceptor (Table 9, code 15) with the best s-score dock pose does not represent the pose with the best non-covalent interactions. Instead, dock pose 3 obtained the best E_refine energy at −14.7028 kcal/mol and the best non-covalent interaction Figure 7 at −12.9233 kcal/mol Table 13.

**Figure 8:**
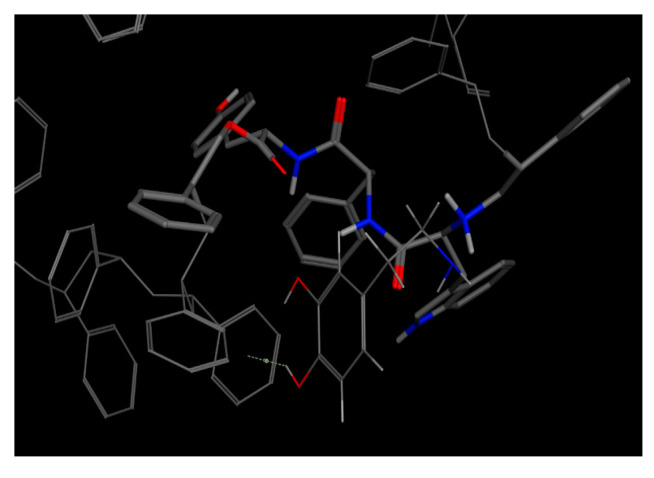
Best docking score results of the dopamine–WFT complex (docked with MOE).

In contrast to the other two bioreceptors, the best dock pose for the WFT bioreceptor (Table 9, code 19) obtained the best non-covalent interactions with tryptophan, phenylalanine, and threonine as seen in Figure 8 and the best E_refine at −15.2311 kcal/mol Table 14.

From the parameters calculated with MOE, the bioreceptor with the best E_refine energy is SD followed by WFT. Although the E_refine parameter is not a ∆G binding free energy calculation, it is possible to use it as an important parameter to study ligand–receptor affinity without resorting to ∆G binding free energy estimations.

### 2.4. Interaction Distances

Another criterion used to assess the docking results was through a graphical interface that displays the calculated interaction models [42]. This visualization was conducted using the PyMOL software for both the DAT and the three selected bioreceptors [43].

**Figure 9:**
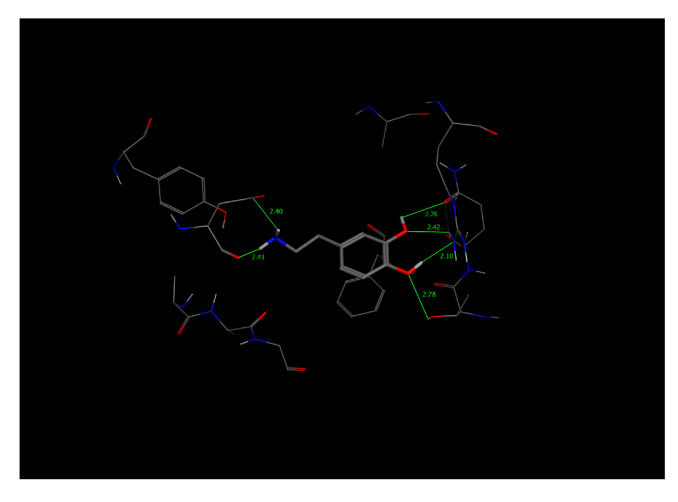
Visualization of the interaction between the DAT and DA. The length of the hydrogen bonds formed (Green dotted line) are shown.

Figure 9 presents the possible interactions through hydrogen bonds (green dotted lines) that were simulated to calculate the affinity between the DAT and DA. The distances of these hydrogen bonds are shown in the image. Only four possible interactions were evaluated because the hydroxyl groups in the ring were closer to the protein. The results yielded two of 2.8 Å, one of 2.7 Å, and one of 2.4 Å.

**Figure 10:**
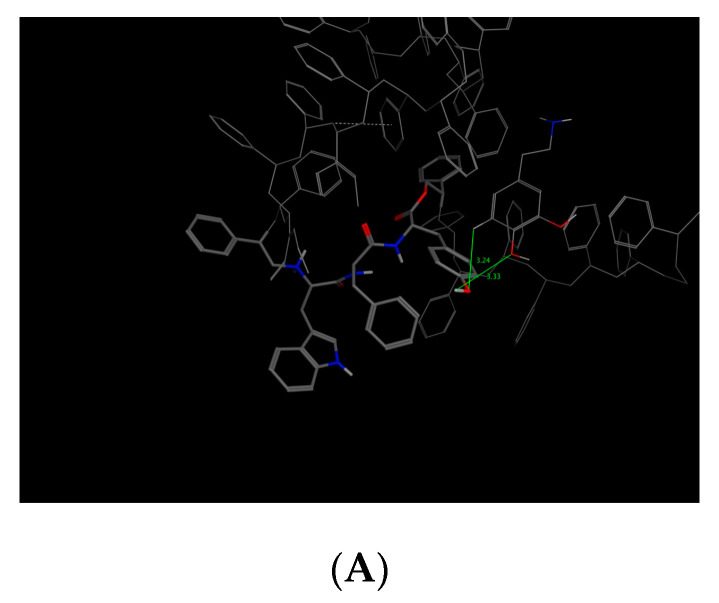

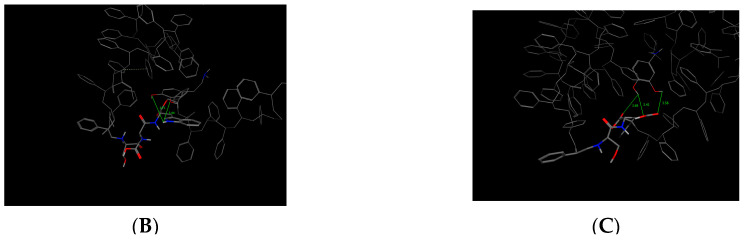
Visualization of the interactions of the selected bioreceptors showing the measured hydrogen bonds formed between dopamine and the amino acids of the bioreceptor. (**A**) Bioreceptor WFT. (**B**) Bioreceptor SDW. (**C**) Bioreceptor SD.

With this criterion, we determined whether the distances of the selected bioreceptors were similar to those of the DAT. The images corresponding to the results of the docking for bioreceptors SD, SDW, and WFT are denoted in Figure 10.

As presented in Figure 9, the length of the interactions created between DA and each of the bioreceptors can be observed. These results are further summarized in Table 15, wherein bioreceptors SDW and WFT exhibit distances greater than the distances reported for the hydrogen bonds formed between the DAT and DA, although they have better interaction energies in terms of affinity. Conversely, for bioreceptor WFT, the distance of one of the hydrogen bonds between the bioreceptor and DA is 3.3 Å. Therefore, the distance is decreased by one tenth when compared against the shortest bond that can be formed with the DAT according to the simulations. However, this bioreceptor does not show a higher affinity than the one from DA reuptake.

Based on these results, three of the designed bioreceptors were candidates to be synthesized for in vitro tests to assess their affinity for DA, and their selectivity and stability as a functional part of a biosensor.

However, the affinity of the bioreceptors for the catecholamines epinephrine and norepinephrine was evaluated, because the structural similarity of these catecholamines could interfere with the recognition and subsequent quantification of dopamine, as shown in Table 16.

Based on the binding affinity obtained, it is clearly evident that bioreceptors have a greater affinity for dopamine than for the other catecholamines. Additionally, the WFT bioreceptor has a slightly larger difference with respect to norepinephrine and epinephrine, which suggests that it may have greater selectivity for dopamine.

In brief, two of the bioreceptors report better DAT interaction energies, and both are formed by three amino acids and are polymerized at the C and N terminals with polystyrene. The third bioreceptor has shorter distances for interaction with DA, although the interaction energy was –0.1 kcal/mol, which is weaker than the energy reported for the DAT.

## 3. Materials and Methods

### 3.1. Enzyme and Receptor Analysis

As described before, tyrosinase has been previously used to design DA biosensors because it is active in vivo. This enzyme catalyzes the hydroxylation and oxidation of diphenols to o-quinones in the presence of oxygen [44], and this reaction can be measured with electrodes that monitor the reduction in catecholamines [13].

Hence, the group to which tyrosinase belongs to was identified as 1.14.18 according to the Nomenclature Committee of the International Union of Biochemistry and Molecular Biology (NC-IUBMB). This group represents the class of oxidoreductases EC 1, with a subclass that acts by donating electron pairs with the incorporation or reduction in molecular oxygen [14], and with a subclass that uses another compound as a donor and incorporates an oxygen atom [18,45]. There are eight different types of tyrosinase in this type of enzyme, all of which are denoted in Table 17.

Conversely, the receptors and enzymes characteristic of DA interactions in biological processes were identified. In the case of DA receptors, the five D1-5 receptors have been described in the literature [10]. 

We also considered the DAT, the protein that modulates the availability of DA in the synaptic cleft, because it is responsible for DA reuptake into the presynaptic neurons [22].

In addition, other proteins that had been crystallized with DA or its analogs in the PDB were evaluated. The amino acid sequences and interaction of these proteins were studied in the PDB, where they were also downloaded in the .pdb format.

### 3.2. Molecular Docking

Molecular docking was carried out in order to calculate the affinity interactions. As part of the procedure used for the simulations in AutoDock Tools (version 1.5.6), the water molecules that could have been included in the protein.pdb file were removed, polar hydrogens were added to the proteins, and the Gasteiger charges were added as previously described [25]. Each analysis grid was a variant and was selected according to the analyzed protein or bioreceptor to verify whether all the amino acids involved in the interaction were being evaluated. The results were analyzed by two criteria. The first criterion was the lowest energy released between the protein–ligand or bioreceptor–ligand complexes because the stability of a complex can be measured by the negative magnitude of the Gibbs free energy [20]. The second criterion was RMSD.

Each calculation was performed thrice, and the averages and standard deviations of the lowest energy value for each protein were obtained.

### 3.3. Free Energy Calculations

Calculations for estimating the ∆G binding free energy of the dopamine-4xp1 protein complex were based on the protocol described in previous articles that focused on estimating ligand selectivity from binding free energy [46]. The topology file for dopamine required molecular dynamic simulations to be carried out, which were generated with SwissParam [47]. The starting complex was built from the best dopamine configuration from the molecular docking performed in Autodock vina. Calculations were carried out in Gromacs 2018.1 series using the CHARMM27 force field [48](see supplementary material for all input files). According to the thermodynamic cycle for free energy calculations, dopamine’s Van Der Waals interactions were decoupled using a linear alchemical procedure with ∆λ =0.1 for Van der Waals and the same ∆λ for Coulombic transformations resulting in 10 λ values (0.0, 0.1, 0.2, 0.3, 0.4, 0.5, 0.6, 0.7, 0.8, 0.9, and 1.0). We obtained a total of 21 different systems that can simulate a complex between dopamine and its receptor, 4xp1 [49]. Using the steepest descent algorithm with 5000 steeps for dopamine and 50,000 for the complex allowed for an energy minimization for each different system to be calculated. The systems’ equilibration was performed in two phases: canonical ensemble (NVT) and isothermal-isochoric ensemble (NPT) to couple temperature and pressure, respectively. The temperature was coupled with 2 ns of Langevin dynamics at 298 K as the reference temperature in the NVT ensemble [50]. The NPT ensemble was performed using the Parrinello–Rahman coupling algorithm at 1 atm. [51]. Data collection was carried out from NPT equilibration under the same conditions of every equilibrium phase for 5 ns. 

### 3.4. Molecular Docking Refining

The force field parameters required to estimate ∆G binding free energy with the previously described pathway for the best average affinity bioreceptors (SD, SDW, and WFT) are still not available; therefore, we decided to validate the results of the molecular docking obtained with Autodock vina using MOE (Chemical Computing Group, v2015.10). Following this methodology, we obtained a relationship between the score function and score energies and the calculated binding free energy with MD, and we extrapolated the energies’ trend to the selected bioreceptors. 

The CHARMM27 force field in MOE was used for parametrizing bioreceptors, protein, and dopamine ligands with a 0.1 gradient RMSD. The initial scoring was estimated with the London ∆G function defined to obtain 30 initial dopamine poses. Afterwards, each binding pose was rescored with the GBVI/WSA ∆G function to obtain a total of 5 dopamine poses. Finally, the best scoring poses with a logical location inside of each bioreceptor and protein were taken as reference to estimate a potential ∆G binding free energy.

### 3.5. Bioreceptor Design

Once the enzyme calculations were carried out thrice, the three enzymes with the best affinity values (kcal/mol) were selected, and the corresponding bioreceptors were designed based on the amino acids that interact with DA. Thus, possible bioreceptors were designed for the three selected enzymes using the ChemDraw^®^ software.

Each designed peptide structure was stored with the. cdmxl extension and was subsequently optimized using the MMFF94s force field in the Avogadro^®^ software tool, with four update steps and using the down-gradient algorithm option [52]. The bioreceptor minimum energy check was verified by means of the Avogadro graphic interface when it reached 0.0000. The. pdbqt files were generated after saving each bioreceptor. The description for this procedure is found in previous studies [52,53].

We selected this software and this molecular energy and geometry optimization algorithm because they allow global minimum to be reached instead of a partial value through convergence toward the minimum, wherein the forces must reach the zero value and where the accepted threshold is 4.5 × 10^−4^ [54]. However, it should be considered that important parameters, such as the speed of convergence, stability, and computational cost, can deteriorate when processing some of the bioreceptors because as the number of links increases as well as the link and torsion angles, the difficulty and time for molecular geometry to converge also usually increase [54].

The designed bioreceptors, based on the amino acids of the enzymes involved in the DA recognition process, were evaluated based on an adapted Trott and Olson methodology (2009). Since the designed bioreceptors were built in silico by the authors instead of obtaining them from the PDB, the procedure bypassed the pretreatment step that adds polar hydrogens to the ligand and conversion of the file to .pdbqt format because this is the original extension with which they were created.

The previous section describes how the bioreceptor affinities were calculated as per the adapted Trott and Olson methodology (2009). Structural modifications were also applied to each designed bioreceptor to achieve interaction energies similar to those reported for the enzymes. These variations are presented below:

1. Amino acid sequences (i.e., AGA).
2. Amino acid sequence with intermediate methylene bonds (i.e., A- (CH2) n-G-A).
3. Amino acid sequences polymerized at the N and C terminals with both polyethylene and polystyrene (i.e., Poly-AGA-Poly).
4. Amino acid sequences with intermediate methylene bonds, polymerized at the N and C terminals with both polyethylene and polystyrene (i.e., Poly-A- (CH2) n-GA-Poly).
5. Variation of amino acids according to their chemical nature, considering the results of the bioreceptors with the previous modifications.

## 4. Conclusions

An in silico methodology was implemented to design bioreceptors through multiple structural changes, controlling variables such as amino acid sequences according to their quantity, their linking bonds, their stereochemistry, and the classification group and steric volume of the bioreceptor. From this, it was possible to obtain three candidates for later synthesis.

These three bioreceptors, in structural terms, are simpler than a protein, and in this case, the DAT; for example, the DAT contains more than six hundred amino acid residues, whereas the bioreceptors have a maximum of three amino acids. Therefore, miniaturization of the DAT was achieved in terms of recognition and molecular interaction with DA. For the simulated interactions, the interaction energy improved by −0.2 kcal/mol compared with the interaction energy presented by the DAT towards DA. In fact, in one case, it was possible to obtain an interaction distance 0.1–0.5 Å closer than that of DAT.

The first bioreceptor corresponds to code 19 assigned in the paper; it is composed of the polystyrene–WFT–polystyrene sequence, with an interaction energy of −5.4 kcal/mol and an interaction distance of 3.3 Å. The second bioreceptor corresponds to code 15 and is formed by the polystyrene–SDW–polystyrene structure. It presented an affinity of −5.3 kcal/mol for DA and an interaction distance of 3.7 Å. Finally, the third one is composed of two amino acids and corresponds to bioreceptor code 14, with a polystyrene–SD–polystyrene structure, an interaction energy of −5.1 kcal/mol, and a length of interaction with DA of 2.3 Å.

According to the parameters calculated with MOE, the bioreceptor with the best E_refine energy is SD followed by WFT. Although the E_refine parameter is not a ∆G free binding energy calculation, it is possible to use it as an important parameter to study ligand–receptor affinity without resorting to ∆G binding free energy estimations.

Bioreceptor candidates were obtained by implementing computational tools that minimize the trial and error stage of the design, synthesis, and in vitro testing processes.

## Figures and Tables

**Table 1:** Targets related to dopamine that have been crystallized and registered in the Protein Data Bank (PDB).

Common Name	PDB Nomenclature
Crystal structure of human tyrosinase related protein 1	5M8L
The Phenylacetyl-CoA monooxygenase PaaABC subcomplex with phenylacetyl-CoA	4IIT
Structure of *Drosophila* dopamine transporter bound to neurotransmitter dopamine	4XP1
Structure of AED7-norepineprhine complex	3DYE
ABC-transporter choline binding protein in complex with acetylcholine	2RIN
Structure of biogenic amine binding protein from *Rhodnius prolixus*	4GET
Structure of human sulfotransferase SULT1A3 in complex with dopamine and 3-phosphoadenosine 5-phosphate	2A3R
Quinone reductase 2 in complex with dopamine	2QMZ
Structure of the human dopamine D3 receptor in complex with eticlopride	3PBL
Structure of the human D4 dopamine receptor in complex with Nemonapride	5WIU
Crystal structure of the N-terminal domain of DrrA/SidM from *Legionella pneumophila*	3NKU
Structure of human I113T SOD1 mutant complexed with dopamine in the p21 space group	4A7V
Structure of Norcoclaurine synthase from *Thalictrum flavum* in complex with dopamine and hydroxybenzaldehyde	2VQ5
Structure of *Drosophila melanogaster* E47D dopamine N-acetyltransferase in ternary complex with CoA and Acetyl-dopamine	5GIG
Structure of human D-amino acid oxidase complexed with imino-DOPA	2E82

**Table 2:** X-ray resolutions and affinity calculations for dopamine with the different targets.

Target	Interaction Energy (Average) (kcal/mol)	X-ray Resolution
5M8L	−6.2	2.35 Å
4IIT	−6.1	4.30 Å
4XP1	−5.2	2.89 Å
3DYE	−5.6	1.75 Å
2RIN	−5.0	1.80 Å
4GET	−5.4	2.24 Å
2A3R	−6.3	2.60 Å
2QMZ	−5.6	2.10 Å
3PBL	−5.6	2.89 Å
5WIU	−4.5	1.96 Å
3NKU	−5.5	2.10 Å
4A7V	−5.2	1.00 Å
2VQ5	−5.0	2.09 Å
5GIG	−4.9	1.30 Å
2E82	−5.9	2.70 Å

**Table 3:** Interaction energies for the first group of bioreceptors designed based on the amino acid sequences from the hotspots of 5M8L, 4IIT, 2A3R, and 2E82.

Bioreceptor Code	Amino Acid Sequence	Interaction Affinity Average (kcal/mol)	Standard Deviation (kcal/mol)
1.0	NWR	−2.6	0.0
2.0	RQKYSSMMGPSPNKNFI	−3.6	0.0
3.0	FPFDKHEAH	−3.5	0.1
4.0	QHYYG	−2.9	0.0

**Table 4:** Interaction energies for the second group of bioreceptors designed to include methylene bridges within the amino acid sequences from the hotspots of 5M8L, 4IIT, 2A3R, and 2E82.

Bioreceptor Code	Amino Acid Sequence with Methylene Bridges	Interaction Affinity Average (kcal/mol)	Standard Deviation
1.1	N(CH_2_)_4_W(CH_2_)_2_R	−3.0	0.0
2.1	R(CH_2_)Q(CH_2_)_7_KYSS(CH_2_)_10_MM(CH_2_)GP(CH2)SPN(CH_2_)K(CH_2_)N(CH_2_)_5_F(CH_2_)_9_I	−3.1	0.1
3.1	F(CH_2_)P(CH_2_)_2_F(CH_2_)D(CH_2_)_5_K(CH_2_)H(CH_2_)_2_EAH	−3.6	0.1
4.1	Q(CH_2_)_10_H(CH_2_)_2_Y(CH_2_)Y(CH_2_)_7_G	−3.0	0.0

**Table 5:** Interaction energy for the third group of bioreceptors where the N and C terminals of each peptide, with or without methylene bridges, were polymerized with either polyethylene or polystyrene.

Bioreceptor Code	Amino Acid Sequence	Interaction Affinity Average (kcal/mol)	Standard Deviation (kcal/mol)
1.0.1	poly (ethylene)-NWR-poly (ethylene)	−4.0	0.0
2.0.1	poly (ethylene)-RQKYSSMMGPSPNKNFI-poly (ethylene)	−3.7	0.0
3.0.1	poly (ethylene)-FPFDKHEAH-poly (ethylene)	−3.9	0.0
1.1.1	poly (ethylene)-N(CH_2_)_4_W(CH_2_)_2_R-poly (ethylene)	−4.0	0.0
2.1.1	poly(etileno)-R(CH_2_)Q(CH_2_)_7_KYSS(CH_2_)_10_MM(CH_2_)GP(CH2)SPN(CH_2_)K(CH_2_)N(CH_2_)_5_F(CH_2_)_9_I-poly (ethylene)	−4.1	0.1
3.1.1	poly (ethylene)- F(CH_2_)P(CH_2_)_2_F(CH_2_)D(CH_2_)_5_K(CH_2_)H(CH_2_)_2_EAH- poly (ethylene)	−4.4	0.0
1.0.2	poly (styrene)-NWR-poly (styrene)	−5.1	0.1
2.0.2	poly (styrene)-RQKYSSMMGPSPNKNFI-poly (styrene)	−4.8	0.0
3.0.2	poly (styrene)-FPFDKHEAH-poly (styrene)	−4.8	0.1
1.1.2	poly (styrene)- N(CH_2_)_4_W(CH_2_)_2_R-poly (styrene)	−4.4	0.1
2.1.2	poly (styrene)-R(CH_2_)Q(CH_2_)_7_KYSS(CH_2_)_10_MM(CH_2_)GP(CH2)SPN(CH_2_)K(CH_2_)N(CH_2_)_5_F(CH_2_)_9_I-poly (styrene)	−5.2	0.0
3.1.2	poly (styrene)-F(CH_2_)P(CH_2_)_2_F(CH_2_)D(CH_2_)_5_K(CH_2_)H(CH_2_)_2_EAH- poly (styrene)	−5.1	0.1

**Table 6:** Interaction energies of the Polystyrene–NWR–polystyrene bioreceptor, and of each derived bioreceptor after stereochemical modification.

Bioreceptor Code	Stereochemistry	Interaction Affinity Average (kcal/mol)	Standard Deviation (kcal/mol)
1.0.2	SSS	−5.1	0.1
1.0.2.2	RRS	−4.5	0.0
1.0.2.3	RSR	−4.9	0.0
1.0.2.4	RSS	−5.0	0.1
1.0.2.5	SRS	−4.8	0.0
1.0.2.6	SSR	−4.9	0.1
1.0.2.7	RRR	−5.0	0.0

**Table 7:** Interaction energies of the Polystyrene–[G]_n_–polystyrene bioreceptor, and of each derived bioreceptor after varying the number of glycine molecules.

Bioreceptor Code	Peptide	Interaction Affinity Average (kcal/mol)	Standard Deviation
4.0	GG	−4.9	0.0
4.1	GGG	−5.1	0.0
4.2	GGGG	−4.8	0.0

**Table 8:** Interaction energies of the bioreceptors designed from single amino acids.

Bioreceptor Code	Tripeptide	Interaction Affinity Average (kcal/mol)	Standard Deviation (kcal/mol)
4.1	GGG	−5.1	0.0
5	FFF	−5.0	0.0
6	AAA	−4.3	0.0
7	NNN	−4.5	0.0
8	CCC	−4.8	0.0
9	SSS	−4.9	0.0
10	HHH	−4.8	0.1

**Table 9:** Interaction energies of the Polystyrene–[AA]_n_–polystyrene bioreceptors, each with a different combination of amino acids.

Bioreceptor Code	Peptide	Interaction Affinity Average (kcal/mol)	Standard Deviation (kcal/mol)
11	LS	−4.5	0.1
12	RD	−4.7	0.0
13	RDYF	−5.1	0.0
14	SD	−5.1	0.0
15	SDW	−5.3	0.1
16	WFF	−4.6	0.0
17	WFFH	−4.6	0.1
18	WFFN	−5.1	0.0
19	WFT	−5.4	0.0
20	WH	−4.5	0.1
21	WHF	−4.5	0.1
22	YDN	−5.1	0.0
23	YF	−5.0	0.0

**Table 10:** ∆G binding free energy calculated for the dopamine-4xp1 complex.

Protein	PDB	∆G_calc_ (kcal/mol)
Drosophila melanogaster	4xp1	−26.760 ± 0.8 *

* Calculated with the standard formula for adding quantities with error propagation.

**Table 11:** Refined docking results obtained with MOE for the dopamine-4xp1 complex.

Pose Dock	S-Score	Rmsd_Frefine	E_Refine
1	−4.4819	1.7480	−21.4354
2	−4.4353	2.1955	−17.7621
3	−4.3577	2.5360	−15.2682
4	−4.2844	1.6866	−17.8818
5	−4.2734	1.0441	−17.8700

**Table 12:** Refined docking results obtained with MOE for the dopamine–SD complex.

Dock Poses	S-Score	Rmsd_Frefine	E_Refine
1	−4.3424	1.0292	−13.2554
2	−4.1898	2.1788	−17.7353
3	−4.1333	1.3140	−14.0582
4	−4.1281	1.9552	−14.4497
5	−4.0736	1.0255	−14.1685

**Table 13:** Refined docking results obtained with MOE for the dopamine–SDW complex.

Pose Dock	S-Score	Rmsd_Frefine	E_Refine
1	−4.2331	1.0382	−12.9233
2	−3.9911	0.9235	−13.6952
3	−3.9721	1.9986	−14.7028
4	−3.8594	1.6950	−13.3851
5	−3.8188	1.6280	−13.0394

**Table 14:** Refined docking results obtained with MOE for the dopamine–WFT complex.

Pose Dock	S-Score	Rmsd_Frefine	E_Refine
1	−4.1360	1.1994	−15.2311
2	−3.9235	0.9766	−12.6343
3	−3.9108	1.8467	−7.7354
4	−3.9048	1.2430	−13.0115
5	−3.8932	1.5533	−11.9384

**Table 15:** Hydrogen bond distances of each of the analyzed bioreceptors.

Bioreceptor Code	Hydrogen Bond Length (Å)
WFT	3.3
SDW	3.7
SD	3.3

**Table 16:** Evaluation of the affinity of the bioreceptors for catecholamines.

		Dopamine	Norepinephrine	Epinephrine	
	Catecholamines	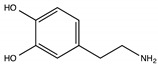	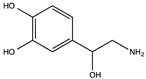	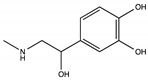	
Bioreceptor					
**SD**		−5.1	−4.6	−4.7	Binding Affinity (Kcal/mol)
**SDW**		−5.3	−4.9	−5.2	
**WFT**		−5.4	−4.6	−4.8	

**Table 17:** Nomenclature of preselected enzymes for analysis.

Systematic Nomenclature	Common Name
EC. 1.14.18.1	tyrosinase
EC. 1.14.18.2	CMP-N-acetylneuraminate monooxygenase
EC. 1.14.18.3	methane monooxygenase (particulate)
EC. 1.14.18.4	phosphatidylcholine 12-monooxygenase
EC. 1.14.18.5	sphingolipid C4-monooxygenase
EC. 1.14.18.6	4-hydroxysphinganine ceramide fatty acyl 2-hydroxylase
EC. 1.14.18.7	dihydroceramide fatty acyl 2-hydroxylase
EC. 1.14.18.8	7α-hydroxycholest-4-en-3-one 12α-hydroxylase
EC. 1.14.18.9	methylsterol monooxygenase
